# Prognostic Factors for Leptospirosis Infection Severity

**DOI:** 10.3390/tropicalmed8020112

**Published:** 2023-02-11

**Authors:** Surangrat Pongpan, Pantitcha Thanatrakolsri, Supa Vittaporn, Patcharin Khamnuan, Punnaphat Daraswang

**Affiliations:** 1 Faculty of Public Health, Thammasat University, Lampang 52190, Thailand; 2 Buriram Hospital, Muang, Buriram 31000, Thailand

**Keywords:** prognostic factors, severe leptospirosis, acute renal failure, respiratory failure

## Abstract

Background: Leptospirosis is an important health problem in Thailand. People infected with leptospirosis may not have any mild symptoms, whereas some people have acute and severe illnesses. It is crucial to strengthen critical patients’ diagnosis and treatment to prevent severe complications and reduce mortality. This study was performed to explore a set of parameters for the prediction of severe leptospirosis illness under routine clinical practice. Methods: A case-control study was conducted in eight general hospitals in Thailand. Retrospective collection data were used, and key information was retrieved from inpatient medical files. Patients were grouped into two severity categories, severe and non-severe infection. Backward elimination was used to reach the final multivariate model. Results: The six significant predictors identified in the study were hemoptysis (OR = 25.80, 95% CI 5.69, 116.92), hypotension (blood pressure < 90/60 mmHg) (OR = 17.33, 95% CI 6.89, 43.58), platelet count < 100,000/µL (OR = 8.37, 95% CI 4.65, 15.09), white blood cell count (WBC) > 14,000/µL (OR = 5.12, 95% CI 2.75, 9.51), hematocrit ≤ 30% (OR = 3.49, 95% CI 1.61, 7.57), and jaundice (OR = 3.11, 95% CI 1.71, 5.65). These predictors could correctly predict the severity of leptospirosis infection in 91.31% of the area under the receiver operation curve (AuROC). Conclusions: The results of this study showed that severe leptospirosis infections have identifiable predictors. The predictors may be used to develop a scoring system for predicting the level of severity.

## 1. Introduction

Leptospirosis is an emerging zoonosis of global importance and manifests with a wide clinical spectrum. Most cases are asymptomatic or mild and self-limited, with some progressing to severe and potentially fatal [1,2]. leptospirosis is more common in tropical or sub-tropical areas. Severe symptoms can include jaundice, renal failure, hemorrhage (especially pulmonary), aseptic meningitis, cardiac arrhythmias, pulmonary insufficiency, and hemodynamic collapse. The case-fatality rate of severe leptospirosis rises above 50% [3]. The prognosis of leptospirosis depends on the severity, early diagnosis, and prompt intensive treatment. If not treated, the patient could develop kidney failure, meningitis, liver damage, and respiratory distress. In some cases, death occurs [4]. 

In Thailand, the morbidity rate of leptospirosis was from 3.26 to 7.76 per 100,000 population from 2010 to 2019. The mortality rate ranged from 0.87% to 2.49%. Most of the patients were aged 45–54 years, followed by 35–44 years, and 25–34 years. The studies presented that their occupation was mainly farming. The most prevalence was found in the Southern Region and followed by the Northeast, the North, and the Central Regions. However, the proportion of patients in the Southern Region has increased more than last year, from 36.5% to 55.6% [5]. 

The symptoms are diverse, typically include fever, and are similar to those of other infectious diseases, such as the common cold, dengue fever, acute fever, headache, and muscle pain. Sometimes patients take medicine; symptoms may be improved until they progress to the second stage of the disease and are severe symptoms or complications [6]. Traditionally, severe leptospirosis, called Weil’s syndrome, has been reported in approximately 5% to 10% of leptospirosis infections [7]. Early treatment may decrease the severity and duration of the disease. In patients with a high clinical suspicion of leptospirosis, initiating antibiotic treatment as soon as possible without waiting for laboratory results is recommended [3].

Previous studies have reported prognostic factors for predicting the severity of disease, including abnormal lung sound [8,9,10,11], hepatomegaly [8], oliguria [9,10,11], consciousness disorders [10], jaundice or icterus [10,12,13], hypotension [8,9], systolic blood pressure ≤ 100 mmHg [11], hemoptysis [12], acute kidney injury (AKI) [14,15], low platelet levels [12,14,15], high leukocytosis level [8,12], low hematocrit [8,12], high serum ALT and AST level [8,10,15], delayed treatment more than 2 days after the onset of illness [10,14,16,17], a history of high blood pressure [10], chronic alcoholism [10], current cigarette smoking [14], male [16], age > 36 years [17], and shock [18]. All studies reported varying prevailing epidemiology and microbiology for each factor.

This study was performed to explore a set of parameters for predicting severe leptospirosis illness in adult patients. Thailand is predominantly an agricultural country, located in a tropical zone; the country has a hot and humid climate which has different epidemiology from other countries. Because the transmission and clinical presentations of leptospirosis vary in different environmental and socioeconomic situations areas [19], predictors of severe outcomes must be evaluated in each epidemiological setting to consider the peculiarities of the region. In the present study, we performed a multicenter observational study to identify prognostic factors of severe leptospirosis. We selected predictor variables using simple clinical laboratory data in routine practice obtained on the day of admission, including demographic, clinical presentation, hemodynamic, hematological, and biochemical laboratory. The predictors will be used to develop a scoring system for predicting the level of severity during the early stages of illness in the next step. Such tools could improve clinical practice by decreasing unnecessary hospitalizations, utilizing limited hospital resources, and improving physicians’ capability in community or general hospitals to make a more accurate early detection. 

## 2. Materials and Methods

### 2.1. Study Design and Study Areas

A case-control study in prognostic research conducted by retrospective data collection. The medical records of patients with leptospirosis were registered between January 2017–December 2020. We performed the study at eight provincial hospitals located in the Northeast and Southern region of Thailand, including Loei hospital, Yasothon hospital, Buriram hospital, Roi Et hospital, Krabi hospital, Ranong hospital, Trang hospital, and Phatthalung hospital.

### 2.2. Study Population 

Patients diagnosed with leptospirosis aged >15 years were confirmed by laboratory tests and admitted to general or central hospitals as mentioned previously. 

#### 2.2.1. *Inclusion Criteria:* Leptospirosis patients who Were Diagnosed According to the Diagnostic Criteria of the World Health Organization (WHO)

*The Diagnostic Criteria of Leptospirosis (The World Health Organization (WHO)* [20].

*Clinical Criteria for Diagnosis*

- Acute febrile illness, chill with headache, and prostration associated with any of the following symptoms including myalgia, conjunctival suffusion, meningeal irritation, anuria or oliguria and/or proteinuria, jaundice, hemorrhages (from the intestines; lung bleeding is notorious in some areas), cardiac arrhythmia or failure, skin rash;
- A history of exposure to infected animals or an environment contaminated with animal urine;
- Other common symptoms include nausea, vomiting, abdominal pain, diarrhea, and arthralgia.

*Laboratory Criteria for Diagnosis*

- Screening test positive in one way or another (Latex agglutination test (LA), Dipstick test, Immuno chromatography test (ICT), ELISA test for leptospirosis positive);
- Confirmatory test positive in one way or another (Culture of Leptospira from blood, cerebrospinal fluid (CSF), or urine, Polymerase Chain Reaction (PCR), Microscopic agglutination test (MAT); single serum sample > 1:400/paired sera: four-fold rising, Immunofluorescent antibody test (IFA); single serum: sample IgM > 1:400/paired sera: a four-fold or greater increase in IgM and IgG antibody titers).

#### 2.2.2. Exclusion Criteria

Patients with co-infection, thalassemia, liver disease, chronic kidney disease, and patients who are missing medical records.

### 2.3. Clinical Definition

Severe leptospirosis was defined by serological confirmation and based on WHO clinical criteria with the presence of at least one of the following criteria: serum creatinine > 3 mg/dL, respiratory failure, and death [12,17].

### 2.4. Study Size Estimation

We calculated the sample size based on a multivariate logistic regression analysis of the predictive factor for severe leptospirosis from the previous study [8]. This calculation gives 90% statistical power at the 5% significance level, two-sided test, a ratio of sample size 1:2 with a proportion of leukocytosis of 58% for the severe group compared with 42% in the non-severe group, the number of patients were 162 and 324, respectively. 

The patients were classified into two groups: the severe group and the non-severe group.

Case (severe group): Patients defined by the WHO case definition of leptospirosis with at least one positive laboratory test and the presence of renal failure (serum creatinine > 3 mg/dL) and/or respiratory failure, death. 

Control (non-severe group): Patients defined by the WHO case definition of leptospirosis with at least one positive laboratory test did not develop any of the symptoms of severe leptospirosis listed above.

### 2.5. Patients and Data Collection 

**Figure 1:**
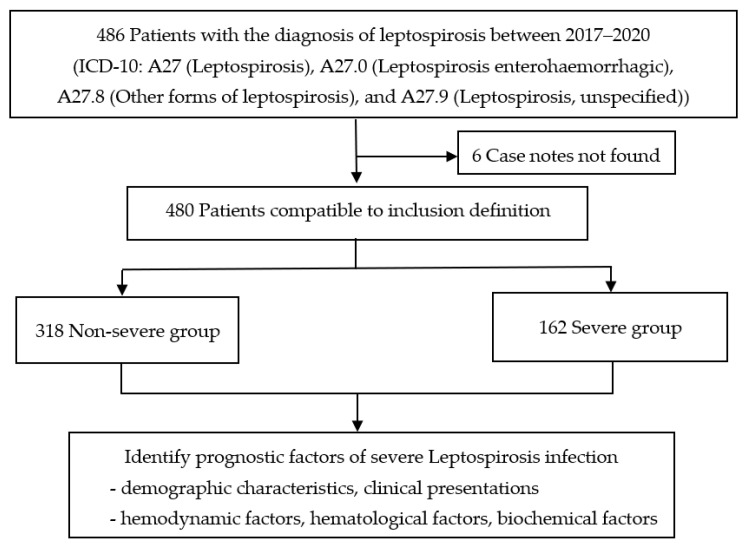
Study flow diagram.

During the study period, a total of 486 patients with confirmed leptospirosis were recruited. Six patients were excluded due to case notes not being found, and 480 patients were included and compatible with the inclusion definition. Of these patients, 318 (66.3%) were non-severe cases, and 162 (33.7%) were severe cases. All demographic data, clinical presentations, hemodynamic factors, and routine laboratory tests were recorded (Figure 1).

In routine clinical practice, the traditional question of the physician is, “What prognostic indicators can be used to classify the leptospirosis infected patients into non-severe and severe groups?” This classification will affect the management of patients. In the non-severe group, outpatient close follow-up and proper education might be appropriate. In the severe group, hospitalization for disease monitoring and the proper treatment will be required.

This study is under the philosophical context of prognostic prediction research. Determinants or causes of disease outcome can be called prognostic factors and aimed to explore prognostic factors of leptospirosis infection severity. The severity of leptospirosis infection (interesting outcome) was categorized into two groups. An occurrence relation can be written as follows.

Design of occurrence relation: outcome = f (determinant x’s)

Leptospirosis infection severity = f (demographic characteristics + clinical presentation + hemodynamic factors + hematological factors + biochemical factors)

*Demographic characteristics*, *clinical data*, *and hemodynamic factors* including gender, age, smoking, alcohol, previous medical history, fever, headache, myalgia, vomiting, cough, diarrhea, abdominal pain, calf tenderness, jaundice, oliguria, anuria, dark yellow urine, redness of the conjunctiva, rash, hemoptysis, systolic blood pressure, and diastolic blood pressure within the first day of admission. 

*Simple laboratory tests: hematological factors and biochemical factors* including hematocrit, white blood cells, platelets, PMN (neutrophil), AST (Aspartate aminotransferase), ALT (Alanine aminotransferase), serum bicarbonate, total bilirubin, and serum creatinine within the first day of admission.

### 2.6. Data Analysis

All data such as the baseline characteristics of the patients, clinical presentations, and simple laboratory tests were entered and recorded in an Excel spreadsheet. Numerical variables were presented with mean and standard deviation (SD) for normally distributed data. The median and interquartile range (IQR) were used for non-normally distributed data. Categorical variables were expressed as numbers and percentages. Patients’ characteristics were compared between groups using an exact probability test for categorical variables and t-tests or the Mann–Whitney U test for numerical variables. A *p*-value < 0.05 was considered significant. In order to identify predictors of severe leptospirosis infection, univariable analysis was performed. All variables were transformed into binary form. Continuous variables were also categorized into two binary forms. Cut-off points developed from clinically meaningful, statistically significant, using the LOWESS method and AuROC to identify the appropriate cut-off point to separate the non-severe and severe disease. Variables with a *p*-value < 0.05 from the univariable analysis were then tested in multivariable models using a backward elimination model, in which factors with the largest *p*-value (*p*-value > 0.05) were sequentially deleted until only significant predictors remained in the final model. The ability of prediction was presented with an area under the receiver operation curve (AuROC).

## 3. Results

### 3.1. Patient Characteristics 

We studied 480 leptospirosis patients in the development data set. Adult patients with leptospirosis infection were categorized as non-severe (n = 318) and severe groups (n = 162). Baseline characteristics, clinical presentation, hemodynamic, hematological, and biochemical laboratory data were observed at the time of admission. The overall mean age was 43.2 years, most individuals were male. The most common symptoms in both groups were fever (100%), and headache (100%), among the severe group, 35 (21.6%) had diarrhea and abdominal pain, 71 (43.8%) had jaundice, 12 (7.4%) had oliguria, 25 (15.4%) had anuria, 46 (28.4%) had hemoptysis, and twelve of these severe cases (7.4%) died. The mean systolic blood pressure (SBP) and diastolic blood pressure (DBP) in the severe group were lower than in the non-severe group (98.9 ± 23.8 vs. 116.7 ± 16.7 mmHg and 59.6 ± 16.0 vs. 69.5 ± 11.1 mmHg, respectively). Laboratory results displayed that hematocrit, platelets, and bicarbonate were lower in severe cases, while white blood cells (WBC), neutrophils, aspartate transaminase (AST), alanine transaminase (ALT), total bilirubin, and creatinine were higher. Comparison between the severe and non-severe groups showed similar demographic data, clinical characteristics, and laboratory results, including gender, age, smoking, alcohol drinking, previous medical history, fever, headache, myalgia, vomiting, cough, calf tenderness, dark yellow urine, redness of the conjunctiva, and rash. The differences between groups included diarrhea (*p* = 0.007), abdominal pain (*p* = 0.026), jaundice (*p* < 0.001), oliguria (*p* < 0.001), anuria (*p* < 0.001), hemoptysis (*p* < 0.001), SBP (*p* < 0.001), DBP (*p* < 0.001), hypotension (blood pressure < 90/60 mmHg) (*p* < 0.001), hematocrit (*p* < 0.001), WBC (*p* < 0.001), platelet (*p* < 0.001), neutrophils (*p* < 0.001), AST (*p* < 0.001), ALT (*p* < 0.001), bicarbonate (*p* < 0.001) and total bilirubin (*p* < 0.001) (Table 1).

### 3.2. Predictors of Severe Leptospirosis Infection

In order to identify predictors of severe leptospirosis infection, univariable analysis was performed. Potential prognostic factors in this study were male gender, age, diarrhea, abdominal pain, jaundice, hemoptysis, hypotension (systolic blood pressure < 90 mmHg and diastolic blood pressure < 60 mmHg), hematocrit, white blood cell count, platelet count, and total bilirubin. Male gender and age were decided prior to include in the final model whether or not these variables were significantly associated with severe leptospirosis infection at the *p*-value of <0.05 since there were several supported studies that indicated that these variables were significantly associated with severity. All variables were transformed into binary form. Continuous variables were also categorized into two binary forms using the cut-off points developed from clinically meaningful and statistical significance using the LOWESS method and area under the receiver operation curve (AuROC). 

After categorizing the variables, univariate analysis showed that significant variables associated with severe leptospirosis infection included diarrhea (*p* = 0.006), abdominal pain (*p* = 0.024), jaundice (*p* < 0.001), hemoptysis (*p* < 0.001), hypotension (*p* < 0.001), hematocrit ≤ 30% (*p* < 0.001), white blood cell (WBC) count >14,000/µL (*p* < 0.001), platelet count < 100,000/µL (*p* < 0.001), and total bilirubin > 2.5 mg/dL (*p* < 0.001). Although age and gender were not statistically significant, it is found clinically significant in many studies. We decided to select this variable to include in our model. (Table 2)

**Figure 2:**
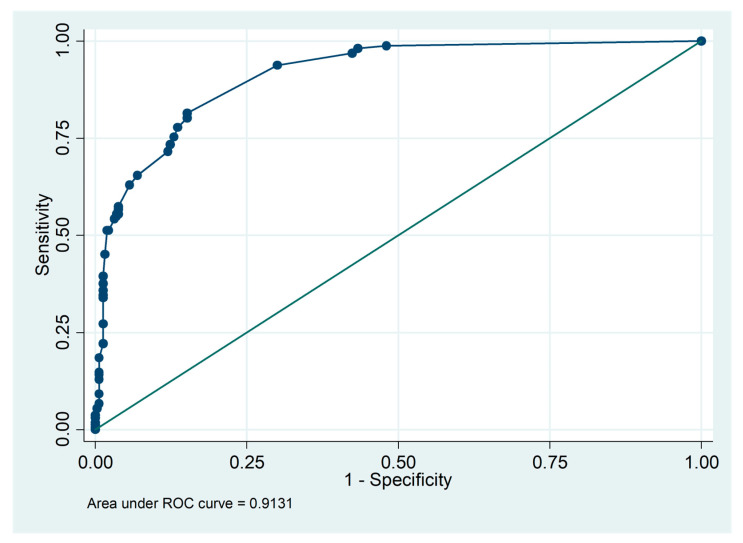
Discrimination of predictors with an area under the receiver operation curve (AuROC).

The next step was the final model building. All factors were used in a full model. Then, we used backward elimination multivariable logistic regression to build the best model that showed these variables in the final model. The six significant predictors identified in the study were: hemoptysis (OR = 25.80, 95%CI = 5.69, 116.92, *p* < 0.001), hypotension (blood pressure < 90/60 mmHg) (OR = 17.33, 95%CI = 6.89, 43.58, *p* < 0.001), platelet count < 100,000/µL (OR = 8.37, 95%CI = 4.65, 15.09, *p* < 0.001), white blood cell (WBC) count > 14,000/µL (OR = 5.12, 95%CI = 2.75, 9.51, *p* < 0.001), hematocrit ≤ 30% (OR = 3.49, 95%CI = 1.61, 7.57, *p* = 0.002), and jaundice (OR = 3.11, 95%CI = 1.71, 5.65, *p* < 0.001). These predictors could predict the severity of leptospirosis infection correctly in 91.31% of the area under the receiver operation curve (AuROC) (Table 3) (Figure 2).

## 4. Discussion

Several predictors for severe leptospirosis have been identified in previous studies. This study was performed to explore a set of parameters for the prediction of severe leptospirosis illness in adult patients. We selected predictor variables by using simple clinical laboratory data in routine practice obtained on the day of admission, including demographic, clinical presentation, hemodynamic, hematological, and biochemical laboratory. The predictors could be different between geographic areas worldwide depending on the socio-demographic parameters, Leptospira species, and serovar.

In our study, the significant predictors identified in the study were hemoptysis, hypotension (blood pressure < 90/60 mmHg), platelet count < 100,000/µL, hematocrit ≤ 30%, jaundice, and white blood cell (WBC) count > 14,000/µL.

### 4.1. Demographic Characteristics

The previous study found that adult males were the principal risk group for leptospirosis [21]. They had clinically more severe outcomes and higher severity of clinical leptospirosis [2,16,22]. Traditionally, this was attributed to the fact that males are usually more exposed to water, and engage in high-risk occupations or recreational activities that predispose them to leptospirosis [23]. In our study, there is no relationship between gender and the severity of the disease, consistent with some previous studies [10,14,24,25,26]. Numerous studies have described age as a predictor of death [18,22,24,25]. Our study, in accordance with Tubiana S. et al., did not identify age as a predictor [14].

A retrospective study found that smoking was a risk factor for alveolar hemorrhage by increasing the permeability of lung capillaries, damaging the alveolar basement membrane, increasing the local inflammatory response, and respiratory distress in severe leptospirosis [27,28]. This study did not identify smoking as a prognostic factor. Few reports have described chronic alcoholism and underlying conditions that are significantly associated with the severity of acute leptospirosis. [10,13]. We did not confirm this in our multivariate analysis as in the previous study [14].

### 4.2. Clinical Data

Leptospirosis patients typically present with fever, headache, myalgia, vomiting, cough, diarrhea, abdominal pain, calf tenderness, oliguria, anuria, dark yellow urine, redness of the conjunctiva, and rash [20]. Previous studies reported that calf tenderness, oliguria, anuria, and conjunctival suffusion were significant predictors of severe leptospirosis [10,18,29]. The results of these studies are different from our study.

### 4.3. Jaundice

Jaundice is a common condition in severe leptospirosis. This was seen in previous studies [10,12,13]. Infection and jaundice (icteric form) were more severe, occurring in 5–10% of all patients. The severity of symptoms may be associated with organ failure. Organs that are most often affected include the liver, kidneys, and central nervous system, and can cause orange skin [20]. Icteric patients had a higher severity and higher risk of death [26].

### 4.4. Hemoptysis

Pneumonia and acute respiratory distress syndrome (ARDS) are the main respiratory symptoms of severe leptospirosis, particularly hemoptysis, which occur in 20–70% [30]. By damaging the endothelial cells of small blood vessels, the glycolipid protein toxin produced by Leptospira then causes severe inflammation of the blood vessels and bleeding. People with severe pulmonary hemorrhage may have severe hemoptysis, which is the leading cause of severe leptospirosis, respiratory failure, and death [20]. In previous studies, hemoptysis was a predictor of severe leptospirosis [12], and a significant predictor of death [31]. In the past report, it was found that hemoptysis was an important clinical symptom to observe. It indicated a major cause of severe pulmonary complications or death [2,32].

### 4.5. Hypotension

Hypotension was found in 60.00% of leptospirosis patients, leading to renal and pulmonary complications. This study found that blood pressure was less than 90/60 mmHg is a predictive factor for severe leptospirosis infection, as in many studies [8,9,11]. Hypotension or cardiovascular collapse on admission was a significant predictor of death [33,34]. Hypotension can be caused by many factors. Patients can develop important hemodynamic abnormalities, secondary to hypovolemia, which is caused by bleeding in various organs, dehydration, and increased capillary permeability from vasculitis due to the direct effects of Leptospira toxins [7,35,36]. Hepatorenal involvements are seen often in moderate and severe leptospirosis due to the pathophysiology of the disease, and damage to the vascular endothelium. A possible explanation for this could be a synergetic effect of the disease (vasculitis) with a hypoxic state, regarding lung involvement and low blood pressure [35].

### 4.6. Simple Laboratory Tests

*Thrombocytopenia (Platelets < 100,000/µL)*

Thrombocytopenia is a frequent complication of leptospirosis, and this condition was present in more than half of patients at the time of hospital admission [37]. This complication is associated with renal failure and is a contributing factor to severe bleeding disorders, which are the leading cause of death [20]. In this study, platelets < 100,000/µL is an independent prognostic factor for severe leptospirosis. This finding is similar to other studies [24,38,39]. The data supported the theory of vasculitis, decreased thrombocyte production, and increased peripheral destruction and consumption of thrombocytes have been considered as potential causes of thrombocytopenia [40].

*Leukocytosis* *(white blood cells ≥ 14,000/µL)*

Higher WBC and neutrophil counts have been demonstrated in patients with severe disease [41]. Leukocytosis occurs in 82% of patients with leptospirosis. White blood cell count above 15,000 was found more frequently in the jaundiced group than in the non-jaundiced group, 58%, and 19%, respectively. Leukocytosis was a significant predictor of death [25,31] due to a larger bacterial inoculum, or inflammatory or immunological reaction in these severe cases [25]. A high total leukocyte count will help in the early detection of severe disease and thus prevent mortality by timely management [42].

*Anemia (hematocrit ≤ 30%)*

*A decrease in hemoglobin levels is a common finding in leptospirosis patients and has been associated with severe disease and poor outcomes* [43,44]*. Anemia was found in severe and dead cases of leptospirosis, probably because endothelial cell damage and occult hemorrhage occurred from lung pathology. Low hemoglobin, a common finding, is caused by several factors including blood loss and hemolysis. Hemorrhage is related to death in patients with leptospirosis* [34]*. Leptospirosis patients with hemorrhage are 71.17 times more likely to die* [18]*; it leads to anemia due to blood loss, and decreased hematocrit. This was found in previous studies* [12,14,15]*. Declining hemoglobin can alert the physician of the possibility that the patient might end up with severe leptospirosis* [42].

There are limitations in our study. Firstly, accurate symptoms and signs might not be captured due to the nature of the retrospective design. Secondly, some variables are various depending on age such as blood pressure or hematocrit; therefore, cut-off points may also be varied. Thirdly, since the clinical data were collected only on the day of admission, we were not able to associate the time period taken to get to the critical illness outcome from the point of enrollment. Further studies should be conducted as a prospective cohort study in order to completely collect useful data.

However, the present study used clinical signs, symptoms, and simple laboratory test values which can be obtained routinely and are usually available in all levels of patient care centers to predict the severity of leptospirosis infection. This scheme may be beneficial to subdistrict hospitals, district hospitals, and provincial hospitals where confirmation is difficult due to the cost of an investigation.

## 5. Conclusions

The severity of leptospirosis infection is significantly associated with some routine clinical parameters. Hemoptysis, jaundice, hypotension (blood pressure < 90/60 mmHg), platelet count < 100,000/µL, white blood cell (WBC) count > 14,000/µL, and hematocrit ≤ 30% are the predictors that increase severe dengue infection. They may guide clinicians to pay some attention to leptospirosis patients with these risks. In the future implications, these predictors may be used to develop a scoring system for predicting the level of severity and managing leptospirosis infection severity early in the course of the illness.

## Figures and Tables

**Table 1:** Demographic and clinical profiles of patients with non-severe and severe leptospirosis.

Patient Characteristics	Severe	Non-Severe	*p*-Value *
	n (%)	n (%)	
Demographic			
Male	143 (88.3)	263 (82.7)	0.141
Age (years), mean (SD)	43.2 (14.4)	43.2 (14.5)	0.991
Smoke	84 (51.9)	158 (49.7)	0.225
Alcohol	81 (50.0)	158 (49.7)	0.913
Previous medical history	27 (16.7)	56 (17.6)	0.899
Clinical presentation			
Fever	162 (100)	318 (100)	1.000
Headache	162 (100)	318 (100)	1.000
Myalgia	121 (74.7)	245 (77.0)	0.572
Vomiting	41 (25.3)	57 (17.9)	0.072
Cough	37 (22.8)	77 (24.2)	0.821
Diarrhea	35 (21.6)	38 (11.9)	0.007
Abdominal pain	35 (21.6)	43 (13.5)	0.026
Calf tenderness	38 (23.5)	56 (17.6)	0.144
Jaundice	71 (43.8)	42 (13.2)	<0.001
Oliguria	12 (7.4)	0	<0.001
Anuria	25 (15.4)	0	<0.001
Dark yellow urine	11 (6.8)	24 (7.6)	0.854
Redness of conjunctiva	18 (11.1)	31 (9.8)	0.636
Rash	5 (3.1)	4 (1.3)	0.173
Hemoptysis	46 (28.4)	6 (1.9)	<0.001
Hemodynamic, mean (SD)			
Systolic blood pressure (mmHg)	98.9 (23.8)	116.7 (16.7)	<0.001
Diastolic blood pressure (mmHg)	59.6 (16.0)	69.5 (11.1)	<0.001
Hypotension (BP < 90/60 mmHg)	58 (35.8)	9 (2.8)	<0.001
Hematological			
Hematocrit (%), mean (SD)	32.7 (6.0)	37.7 (4.8)	<0.001
White blood cells (/µL), median (IQR)	12,200 (8700, 16,700)	9100 (6500, 12,240)	<0.001
Platelets (/µL), median (IQR)	50,500 (23,000, 91,000)	165,500 (99,000, 239,000)	<0.001
Neutrophils (%), mean (SD)	83.7 (8.5)	75.6 (12.9)	<0.001
Biochemical			
AST (U/L), median (IQR)	57 (37, 104)	42 (27, 66)	<0.001
ALT (U/L), median (IQR)	58 (32, 79)	40 (25, 72)	<0.001
Bicarbonate ((mmol/dL)), mean (SD)	19.5 (3.8)	21.7 (3.6)	<0.001
Total bilirubin (mg/dL), median (IQR)	3.3 (1.4, 8.4)	0.9 (0.5, 2.1)	<0.001
Creatinine (mg/dL), median (IQR)	5.1 (3.1, 6.7)	1 (0.8, 1.2)	<0.001
Duration of fever (days), mean (SD)	4.5 (2.0)	4.0 (2.4)	0.031
Duration of admission (days), median (IQR)	6 (3, 9)	3 (2, 5)	<0.001
In hospital dead	12 (7.4)	0	<0.001

* *p*-value from exact probability test, independent *t*-test, or Wilcoxon rank-sum test. BP: Blood pressure; AST: aspartate transaminase; ALT: alanine transaminase.

**Table 2:** Crude odds ratios (OR) and 95% confidence interval (CI) for leptospirosis infection.

Patient Characteristics	Crude Odds Ratio	95%CI of Odds Ratio	*p*-Value
Gender			
male	1.58	0.90, 2.76	0.110
female	1.00	reference	
Age (years)			
<40	1.03	0.70, 1.52	0.871
≥40	1.00	reference	
Diarrhea			
yes	2.03	1.23, 3.36	0.006
no	1.00	reference	
Abdominal pain			
yes	1.76	1.08, 2.89	0.024
no	1.00	reference	
Jaundice			
yes	5.13	3.27, 8.03	<0.001
no	1.00	reference	
Hemoptysis			
yes	13.13	3.81, 45.27	<0.001
no	1.00	reference	
Hypotension			
yes	19.15	9.17, 39.99	<0.001
no	1.00	reference	
Hematocrit (%)			
≤30	7.85	4.34, 14.19	<0.001
>30	1.00	reference	
White blood cells (/µL)			
>14,000	3.11	2.13, 4.77	<0.001
≤14,000	1.00	reference	
Platelets (/µL)			
<100,000	10.74	6.82, 16.92	<0.001
≥100,000	1.00	reference	
Total bilirubin (mg/dL)			
>2.5	4.67	3.03, 7.20	<0.001
≤2.5	1.00	Reference	

**Table 3:** Multivariable odds ratios (OR) and 95% confidence interval (CI) for Leptospirosis infection severity.

Indicators	Multivariable OR * (95% CI)	*p*-Value
Hemoptysis	25.80 (5.69, 116.92)	<0.001
Hypotension (BP < 90/60 mmHg)	17.33 (6.89, 43.58)	<0.001
Platelet < 100,000/µL	8.37 (4.65, 15.09)	<0.001
White blood cells ≥ 14,000/µL	5.12 (2.75, 9.51)	<0.001
Hematocrit ≤ 30%	3.49 (1.61, 7.57)	0.002
Jaundice	3.11 (1.71, 5.65)	<0.001

* Odds ratio from multivariable logistic regression.

## Data Availability

Not applicable.
